# Personalized Activity Recognition with Deep Triplet Embeddings

**DOI:** 10.3390/s22145222

**Published:** 2022-07-13

**Authors:** David Burns, Philip Boyer, Colin Arrowsmith, Cari Whyne

**Affiliations:** 1 Orthopaedic Biomechanics Laboratory, Holland Bone and Joint Program, Sunnybrook Research Institute, Toronto, ON M4N 3M5, Canada; philip.boyer@mail.utoronto.ca (P.B.); colin.arrowsmith@sri.utoronto.ca (C.A.); cwhyne@sri.utoronto.ca (C.W.); 2 Division of Orthopaedic Surgery, Department of Surgery, University of Toronto, Toronto, ON M5S 2E8, Canada; 3 Halterix Corporation, Toronto, ON M5E 1L4, Canada; 4 Institute of Biomedical Engineering, University of Toronto, Toronto, ON M5S 2E8, Canada

**Keywords:** human activity recognition, personalized algorithms, machine learning, time series, triplet neural network, inertial sensors

## Abstract

A significant challenge for a supervised learning approach to inertial human activity recognition is the heterogeneity of data generated by individual users, resulting in very poor performance for some subjects. We present an approach to personalized activity recognition based on deep feature representation derived from a convolutional neural network (CNN). We experiment with both categorical cross-entropy loss and triplet loss for training, and describe a novel loss function based on subject triplets. We evaluate these methods on three publicly available inertial human activity recognition datasets (MHEALTH, WISDM, and SPAR) comparing classification accuracy, out-of-distribution activity detection, and generalization to new activity classes. The proposed triplet algorithm achieved an average 96.7% classification accuracy across tested datasets versus the 87.5% achieved by the baseline CNN algorithm. We demonstrate that personalized algorithms, and, in particular, the proposed novel triplet loss algorithms, are more robust to inter-subject variability and thus exhibit better performance on classification and out-of-distribution detection tasks.

## 1. Introduction

Inertial sensors embedded in mobile phones and wearable devices are commonly employed to classify and characterize human behaviors in a number of applications, including tracking fitness, elder safety, sleep, and others [1,2,3,4,5]. Sensor-based HAR is, furthermore, increasingly being used in clinical settings to assist in monitoring and tailoring rehabilitation and physiotherapy activities of patients [6,7,8]. Improving the accuracy and robustness of the algorithms underlying inertial Human Activity Recognition (HAR) systems is an active field of research.

A significant challenge for a supervised learning approach to inertial human activity recognition is the heterogeneity of data between individual users. This heterogeneity occurs in relation to diversity in the hardware on which the inertial data is collected, different inherent capabilities or attributes relating to the users themselves [9], alterations in the environment in which the data is collected [10], and inconsistent sensor placement. This is particularly important in monitoring rehabilitation activities where patient performance is heavily dependent on clinical condition and level of impairment, resulting in large variance in capacity to reproduce idealized versions of exercises.

Large datasets incorporating the full spectrum of user, device, and environment heterogeneity may be considered in addressing these challenges, however, such an approach presents significant logistical and financial challenges. Further, the devices and sensors on which inertial data is collected continuously evolve over time and it may not be feasible to train generic supervised algorithms that perform equally well in HAR for all users and devices. An alternative is to leverage labeled user-specific data for a personalized approach to HAR.

In this research, we experiment with deep feature representation for personalized HAR, specifically considering (1) extracted features from a neural network classifier and (2) an optimized embedding learned using Triplet Neural Networks (TNN) [11,12]. We compare these to a baseline impersonal neural network classifier, and a personalized engineered feature representation.

Contributions of this study include:

- Presentation and evaluation of novel personalized embedding approaches to HAR that enable rapid and computationally efficient user-specific data characterization and classification.
- Adaptation of the TNN metric learning methodology into this personalized user-specific HAR classification regime.
- Extension of the proposed approaches to unseen classes and out-of-distribution (OOD) detection. We illustrate how the personalized methodologies presented in this work are easily extensible to the preceding tasks and are able to achieve high accuracy on the experimental datasets.

The remainder of this paper is organized as follows. We provide a brief synopsis of related work to the topic of personalized approaches to HAR. Section 2 describes the key methodologies proposed, including personalized feature extraction methods and a detailed explanation of the personalized TNN approach. We also describe therein the core model used in the analysis, the preprocessing pipeline, as well as experimental setup inclusive with evaluated datasets. Section 3 presents experimental results, with discussion of these in Section 4. Lastly, in summarizing the findings of the preceding analysis, Section 5 highlights the significance of this work and potential future related research.

### Related Work

HAR from inertial time series data has classically been conducted using a supervised learning approach with non-neural classifiers, after transformation of the data using an engineered feature representation consisting of statistical, time-domain, and/or frequency-domain transforms [13,14,15,16]. Modern supervised learning approaches using convolutional and or recurrent neural networks are increasingly utilized and have demonstrated improvements in classification accuracy over non-neural models [1,17,18,19,20,21]. Both non-neural and neural network supervised learning models have been applied to personalized activity recognition [22,23,24,25,26,27,28,29].

User-specific supervised learning models can be trained through one of three general schemes. First, a user-specific model can be trained de novo with user-specific data or a combination of generic and user-specific data [30]. This is generally not feasible for neural network approaches that require vast datasets and computational resources for training, but works well for non-neural approaches with engineered features [22]. Second, model updating (online learning, transfer learning) with user-specific data is feasible for both non-neural [23,24,25] and neural network supervised learning algorithms [26,29]. Rokni et al. [26] trained a generic convolution neural network architecture and adapted it to specific users by retraining the classification layer while fixing the weights of the convolutional layers with excellent results. A third scheme involves using classifier ensembles [27,28]. Hong et al. [28] trained non-neural models on subpopulations within the training set, and selected user-specific classifier ensembles based on testing the pre-trained classifiers on user-specific data. These personalized methods have all produced favorable results in comparison to generic models. However, generating, validating, and maintaining user-specific supervised learning models presents its own logistical challenges in a production environment. There are also currently regulatory barriers to such an approach in the context of software as a medical device [31].

An alternative approach to personalized activity recognition is to store an embedding of labeled user-specific data. The embedding process performs a feature extraction transformation of data into a new predetermined feature space. The embedding process can be specified a priori with engineered features, and/or be learned from the data (e.g., a deep learning model). The advantage of this methodology is that the embedding method can be fitted or trained to a large dataset in advance, while user-specific interrogation can be rapidly accomplished in a de novo fashion via feature extraction through the pre-trained embedder, with the resulting embedding subsequently used to perform classification and/or characterization. Further benefits of this approach include the capacity to incorporate novel activity classes without model re-training, and identify out-of-distribution (OOD) activity classes (i.e., samples drawn from class distributions previously unseen in classifier training), thereby supporting an open-set activity recognition framework [32,33,34].

The penultimate feature layer of neural network classifiers in various domains have been shown to be useful for classification and other tasks (e.g., visualization, clustering) [35,36]. Sani et al. [36] demonstrated that features extracted from a deep convolutional neural network are superior for generic activity recognition in comparison to engineered features with non-neural models. However, features extracted from deep neural networks are often treated as a side effect of the classifier training, rather than being explicitly sought. Metric learning methods, such as Siamese Neural Networks (SNN) [37] and Triplet Neural Networks (TNN) [11,12,38] optimize an embedding directly for the desired task. Triplet selection strategies have been proposed for domain-specific tasks, which improve performance from the naive implementation. Khaertidnov et al. proposed triplet batch construction based on subject and class distance with attention [39]. In the work by He et al. triplets were sampled based on a hierarchical strategy in the application of fine-grained image classification, where a convolutional neural network was trained to extract low-level features [40]. Inter-class subject variability may also be approached as a domain adaptation problem as in the work by Hao et al. [41], where a domain-invariant deep feature extractor is combined with task-specific networks for the domains of subjects and devices.

## 2. Materials and Methods

### 2.1. Fully Convolutional Neural Network Architecture

The deep learning model architecture adopted in this analysis is the fully convolutional network (FCN) as proposed by Wang et al. [17]. This architecture is considered a strong baseline for time series classification even in comparison to deep learning models with modern architectural features used in computer vision such as skip connections. The FCN model used in this study consists of 3 1D convolutional layers, with rectified linear unit (ReLU) activation, and batch normalization after each layer. Regularization of the model is achieved using dropout applied at each layer. Global average pooling is used after the last convolutional layer to reduce the model sensitivity to translations along the temporal axis, as this ensures the receptive field of the features in the penultimate feature layer includes the entirety of the window segment. The receptive field of filters in the last convolutional layer prior to global average pooling was 13 samples, which is equivalent to 260 ms at a sampling rate of 50 Hz. An $L^{2}$ normalization is applied after global pooling to constrain the embedding to the surface of a unit hypersphere, which improves training stability. Gradient norm clipping to 1.0 is used to mitigate exploding gradients. The impersonal fully-convolutional neural network (FCN), personalized deep feature (PDF), and personalized triplet network (PTN) models described in subsequent sections all use this FCN core architecture.

### 2.2. Feature Embedding Methods

#### 2.2.1. Engineered Features

We use an engineered feature representation to serve as a baseline personalized classifier model. The representation consists of typical statistical and heuristic features used for inertial activity recognition [15], including mean, median, absolute energy, standard deviation, variance, minimum, maximum, skewness, kurtosis, mean spectral energy, and mean crossings. The features are individually computed for each of the data channels in the dataset. All features are individually scaled to unit norm and zero mean across the training dataset.

#### 2.2.2. Deep Features

In addition to engineered hand-crafted features, we train a model to learn time series features directly from the data. A deep feature representation model is created by training an FCN classifier model that consists of the FCN core with a final dense layer with softmax activation. This model is used to directly classify time series segments at test time, and is referred to as the “Impersonal FCN” in this study.

Alternatively, the FCN can also be used at test time to create deep features, or embeddings, for time series segments. Embeddings are created by taking the normalized output from the global average pooling layer (before the fully-connected layer) of the FCN for a given segment. Embeddings are created for a set of reference samples and a set of test samples from the same patient. Inference is then performed using a k-NN search of the reference samples closest to the given test sample. When used in this configuration we refer to the model as a personalized deep feature (PDF) model.

#### 2.2.3. Triplet Network Features

While training an FCN for direct classification can lead to effective feature representation layers, the distances between samples in that feature space is not explicitly learned. The role of the triplet neural network is to learn an embedding f(x), for data x into a feature space $R^{d}$ such that the Euclidean distance between datum of the same target class (*y*) is small and the distance between datum of different target classes is large. With a squared Euclidean distance metric, triplet loss ($L_{T}$) is defined by Schroff et al. [11] as:

(1) $$L_{T}=\sum_{i}^{T}\max\left\{\left[∥f\left(x_{i}^{a}\right)-f\left(x_{i}^{p}\right){∥}_{2}^{2}-{∥f\left(x_{i}^{a}\right)-f\left(x_{i}^{n}\right)∥}_{2}^{2}+\alpha \right],0\right\}$$

where $x_{i}^{a}$ is a sample from a given class (anchor), $x_{i}^{p}$ is a different sample of the same class (positive), and $x_{i}^{n}$ is a sample of a different class (negative). α is the margin, which is a hyperparameter of the model defining the distance between class clusters. The same embedding f(x) is applied to each sample in the triplet, and the objective is optimized over a training set of triplets with cardinality *T*. The number of possible triplets (*T*) that can be generated from a dataset with cardinality *N* is $O(N^{3})$.

In practice, TNNs converge well before a single pass over the full set of triplets [11], and therefore a subset of triplets must be specifically selected from the full set. First, a naive strategy is implemented whereby triplets are randomly selected from *T*, enforcing only no temporal overlap between anchor and positive samples. Next, a triplet selection strategy is implemented where triplets derive their samples from a single subject, which yields a modified triplet loss function:

(2) $$L_{S}=\sum_{s}^{S}\sum_{i}^{T_{s}}\max\left\{\left[∥f\left(x_{s,i}^{a}\right)-f\left(x_{s,i}^{p}\right){∥}_{2}^{2}-{∥f\left(x_{s,i}^{a}\right)-f\left(x_{s,i}^{n}\right)∥}_{2}^{2}+\alpha \right],0\right\}$$

where $x_{s,i}^{a}$ is a segment of a particular activity class for subject *s* (anchor), $x_{s,i}^{p}$ a segment of the same activity class and subject of the anchor (positive), and $x_{n,i}^{a}$ is a segment of a different activity class but from the same subject as the anchor (negative). $T_{s}$ denotes the full set of triplets that may be drawn from a single subject, and *S* is the full set of subjects. This approach reduces the number of possible triplets to O(N). Various other strategies have been used in the computer vision domain to specifically select hard triplets for improving the efficiency of the TNN training [11].

**Figure 1:**
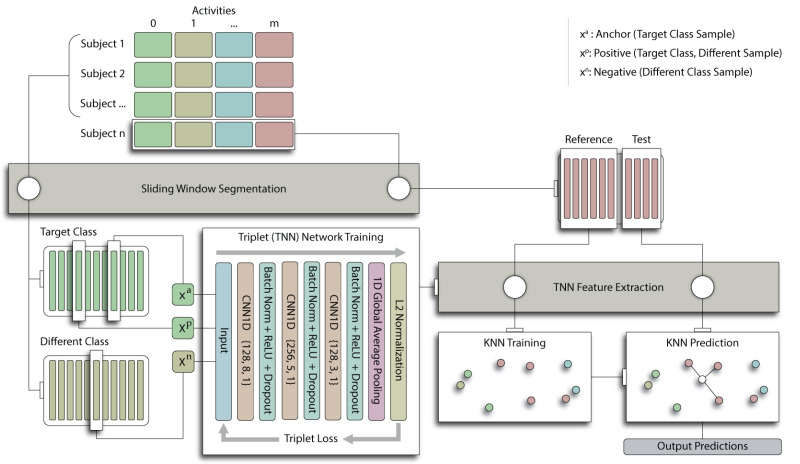
Personalized triplet network (PTN) training and prediction methodology. Beginning from **top left**, each dataset is split into 5 groups for 5-fold cross validation, stratifying the groups by subject. Activity classes are distributed uniformly across groups. Colorization indicates activity classes or model layer as applicable. Sliding window segmentation is then applied to each fold and the segmented test fold is held back. PTN training (**bottom left**) is achieved by drawing two segments $x^{a}$ and $x^{p}$ from the target activity class and one segment $x^{n}$ from a different class, performing a forward pass through the triplet neural network (TNN) for each of the three segments, and computing the triplet loss $L_{T}$. This procedure is then repeated for the set of triplets $T_{i}$ for each activity class *i*. The model is then evaluated by temporal splitting of the test segments for each class into “reference” and “test” sets, ensuring no temporal overlap between reference and test segments. Reference segments from all classes for a given patient are then passed through the TNN and the resulting embeddings are used to train a k-NN model (**bottom right**). Finally, inference is performed by passing test segments though the TNN and performing a k-NN search across the set of reference embeddings.

We derive the PTN embedding f(x) by training the FCN core with triplet loss. In our experiments, we evaluate conventional triplet loss with random triplets (PTN${}^{†}$ as per Equation (1)), and subject triplet loss (PTN as per Equation (2)) with a portion of the triplets being subject triplets and the remainder randomly selected. We use the same optimizer and hyperparameters as for training an impersonal FCN, except the learning rate is reduced to 0.0002 when training the FCN core with triplet loss. The hyperparameter α was initially tuned to a value of 0.3 and kept fixed for all subsequent tests. Despite the greater cardinality of the triplet set, we consistently define an epoch in this manuscript as having *N* samples. At test time, deep features are extracted from reference and test time series segments using the PTN. Inference is then performed using the k-NN approach described in Section 2.2.2. This approach is described in Figure 1.

### 2.3. Data and Preprocessing

Algorithms are evaluated on three publicly available inertial activity recognition datasets: MHEALTH [42], WISDM [43], and SPAR [44]. These datasets encompass a combination of activities of daily living, exercise activity, and physiotherapy activities. Class balance is approximately equal within each and there is minimal missing data. The specific attributes of these datasets are summarized in Table 1.

The MHEALTH data was collected with three proprietary inertial sensors on the subjects’ right wrist, left leg, and chest. The WISDM data was collected from an Android smart watch worn by the subjects, and a mobile phone in the subjects’ pocket. The SPAR data was collected from 20 subjects (40 shoulders) using an Apple smart watch.

The WISDM and MHEALTH data is resampled to 50 Hz, using cubic interpolation, to provide a consistent basis for evaluating model architecture. The time series data are then pre-processed with sliding window segmentation to produce fixed length segments of uniform activity class. A four second sliding window is utilized for the MHEALTH and SPAR datasets, and a ten second window is utilized for WISDM for consistency with previous evaluations [43,44,45]. An overlap ratio of 0.8 is used in the sliding window segmentation as a data augmentation strategy. Engineered feature extraction resulted in 66 features for the WISDM and SPAR datasets, and 174 features for the MHEALTH dataset.

We use only the smart watch data from the WISDM dataset because the smart watch and mobile phone data were not synchronized during data collection. We also exclude four WISDM subjects from the evaluation due to errors in data collection that resulted in absent or duplicated sensor readings (subjects 1637, 1638, 1639, and 1640).

The keras [46] and seglearn [47] open source python libraries were utilized to implement the machine learning models described in this work. The scikit-learn library was used to implement the k-nearest neighbor algorithm.

### 2.4. Experiments

#### 2.4.1. Activity Classification

Classification accuracy is evaluated using five-fold cross-validation grouping folds by subject. Subject distribution across folds is randomized but consistent for each algorithm in keeping with best practices for the evaluation of human activity recognition algorithms [45]. Cross-validated test set performance is summarized for each algorithm on the three datasets in Table 2. Accuracy statistics (mean and standard deviation) are aggregated by subject, not by fold. The statistical significance of performance differences between models is evaluated based on the standard deviation in performance of each model during cross validation. Models were considered significantly different if their mean accuracies were more than two standard deviations apart.

Classification accuracy of the supervised FCN model is tested in addition to three personalized feature classifiers: personalized engineered features (PEF), personalized deep features (PDF), and personalized triplet network (PTN). Inference in the FCN is achieved by taking the direct model prediction for each test segment. The FCN classifier is trained for 150 epochs using the Adam optimizer, categorical cross entropy loss, and a learning rate of 0.001. Inference with the personalized models is achieved by comparing a subject’s embedded test segments to the labeled reference embeddings specific to the subject. For the test subjects, the time series data for each activity is split along the temporal axis, reserving the first 50% for reference data and the latter part for inference. This split is performed prior to sliding window segmentation to ensure there is no temporal overlap of reference and test samples. This partitioning of the data is depicted in Figure 1. To determine the activity class in a test segment, we search the reference embeddings for the three-nearest neighbors (*k*-NN with k=3) using a Euclidean distance metric and a uniform weight decision function.

#### 2.4.2. Embedding Size

A deep feature representation of activity is desirable to minimize the storage and computational cost of personalized feature inference. We assess the effect of embedding size on model performance using five-fold cross validation on the SPAR dataset. For the PDF and PTN models, the embedding size is adjusted at the final dense layer of the FCN core. For the engineered features, we reduce the embedding size by selecting the most important features as ranked using Gini importance [48]. The Gini importance is calculated for the engineered features using an Extremely Randomized Trees classifier [49] with an ensemble of 250 trees.

#### 2.4.3. Reference Data Size

We evaluate the effect of reference data size on model performance, using 50% of the test data as the baseline evaluation. The effect of reference sample quantity on personalized feature classifier accuracy is evaluated using five-fold cross validation on the SPAR dataset. Reference dataset sizes of 4, 8, 16, and 24 segments are tested. The upper bound of 24 segments is constrained by the length of recordings. In each case, the model is tested on the same test set.

#### 2.4.4. Out-of-Distribution Detection

We assess model performance for distinguishing activity classes present in the training distribution from unknown (out-of-distribution) activity classes. This evaluation is performed by training the models on a subset (70%) of the activity classes, and testing with the full set of activity classes in a subject group five-fold cross validation scheme. In each fold, the classes considered out-of-distribution are randomly selected but are consistent across the algorithms evaluated. Out-of-distribution performance is assessed using the area under the receiver operating curve (AUROC) for the binary classification task of in- vs. out-of-distribution.

Out-of-distribution (OOD) classification is implemented for the personalized feature classifiers using a local outlier factor model trained on the in-distribution embeddings on a per-subject basis. The mean distance of the three nearest neighbors is used as the probability output. For the FCN model, we consider the maximum softmax layer output as a confidence measure for the decision function [50].

#### 2.4.5. Generalization to New Activity Classes

Generalization of personalized features to new activity classes is assessed in a manner similar to out-of-distribution detection. Rather than a binary in- vs. out- classification target, each model is trained on data with 30% of the activity classes removed. The model is then tested by performing multi-class classification on the full set of activity classes in the test set, where reference samples for the k-NN are inclusive of the new activity classes. The FCN model is not assessed for this task as generalization to new target classes is not possible due to the static output size of the softmax classification layer. The multiclass classification accuracy is used as the metric for this task.

#### 2.4.6. Computational Expense

Experiments are carried out locally on a computer with two NVIDIA Titan V GPUs for hardware acceleration. Computational expense is evaluated for each model by comparing the fit time, inference time, model size, and reference embedding size with the SPAR dataset on a single fold (test size 0.2). Reference size for personalized feature classifiers is based on single precision 64 feature embeddings, with 16 samples for each of the 7 activity classes.

## 3. Results

### 3.1. Activity Classification

**Figure 2:**
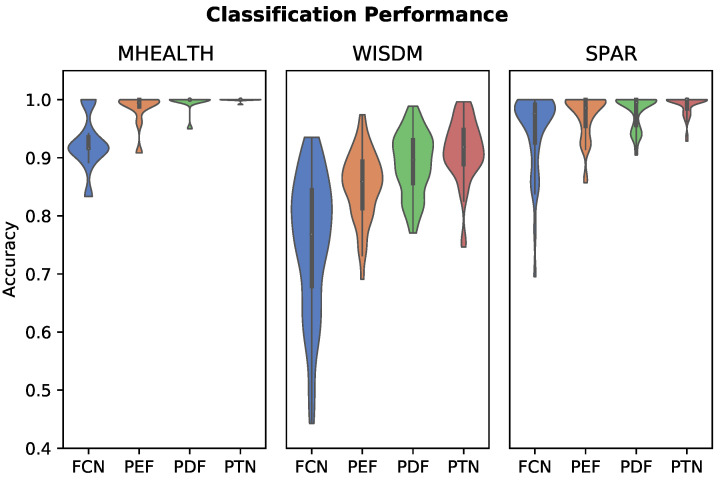
Violin plots showing the distribution of classifier performance by subject using five-fold cross validation. The distributions are cut-off at the minimum and maximum accuracy values. The personalized classifiers have better performance and less inter-subject performance variation than the impersonal FCN (fully convolutional network) model.

Cross-validated test set performance is summarized for each algorithm on the three datasets in Table 2. Accuracy statistics (mean and standard deviation) are aggregated by subject, not by fold. Box and whisker plots demonstrating the variation in performance between individuals are provided in Figure 2.

Personalized feature classifiers out-performed the impersonal FCN classifier and reduced the incidence and degree of negative outlier subjects that exhibited poor performance in the impersonal model. Personalized models reduced inter-subject variability in classification performance. Both the personalized deep feature models (PDF and PTN) outperformed the personalized engineered features (PEF). Specifically, the PTN model utilizing subject triplet loss had the highest classification performance. However, all of the personalized feature classifiers are within one standard deviation of one another. Conversely, the standard deviation of the PTN model is much more constrained around the mean as compared to the other personalized models. Personalized algorithms achieved near 100 percent accuracy for the MHEALTH and SPAR datasets, while the results were significantly lower for WISDM.

### 3.2. Embedding Size

**Figure 3:**
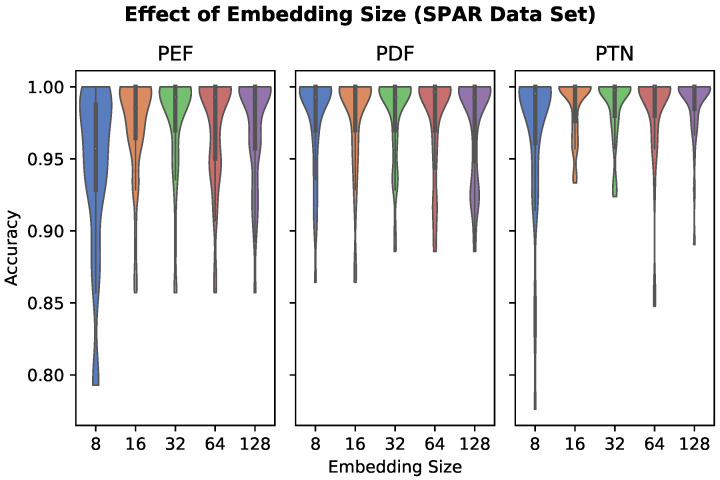
The effect of embedding size (number of features) on personalized feature classifier accuracy, evaluated on the SPAR dataset. The performance of the PEF model appears to degrade at embedding size 16 and below.

Classifier performance as a function of embedding size is plotted in Figure 3. The performance of the PEF model appears to degrade at embedding size 16, with embedding sizes of 8 leading to a significant drop in accuracy.

### 3.3. Reference Data Size

**Figure 4:**
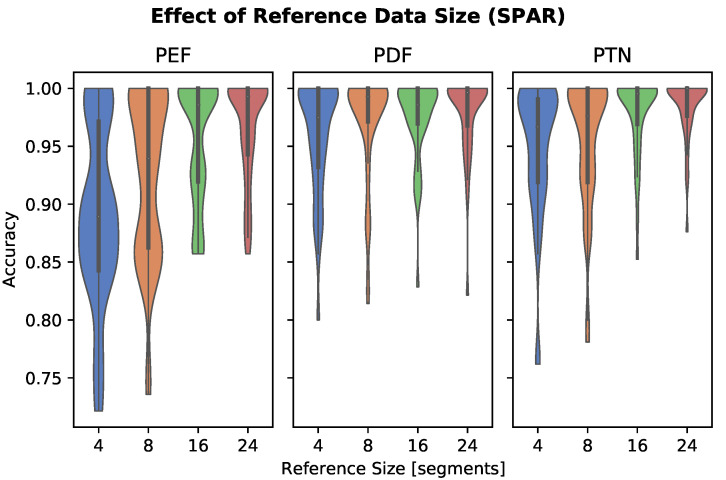
The effect of reference data size (number of reference segments per activity class) on personalized feature classifier accuracy, evaluated on the SPAR dataset. Increasing reference data size results in improved performance for the PEF model. A reference size of four segments results in significantly degraded performance in all models.

Results are plotted in Figure 4. Increasing reference size had a pronounced effect on performance in the PEF model. Reference sizes of eight or more segments resulted in similar performance in the PDF and PTN models.

### 3.4. Out-of-Distribution Detection

**Figure 5:**
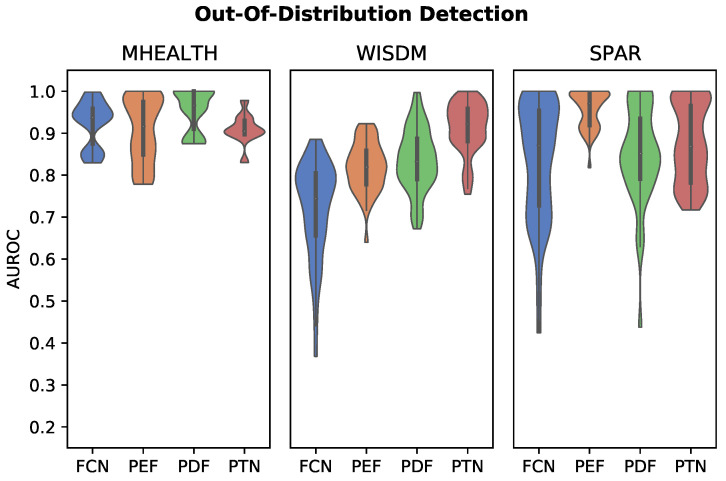
Violin plots showing distribution of OOD detection AUROC across subjects, with 30% of activity classes held back from the training set. The displayed distributions are cut-off at the minumum and maximum AUROC values for each classifier. The PDF, PTN, and PEF classifiers had the highest mean AUROC scores for the MHEALTH, WISDM, and SPAR datasets, respectively.

OOD detection performance is plotted in Figure 5. In contrast to the classification task, the best performing OOD detector appeared to depend on the dataset tested. The PDF, PTN, and PEF classifiers had the highest mean AUROC scores for the MHEALTH, WISDM, and SPAR datasets, respectively. The personalized models achieved AUROCs of greater than 0.8 on each dataset. FCN softmax thresholding, in particular, fared poorly on the WISDM dataset.

### 3.5. Generalization to New Activity Classes

**Figure 6:**
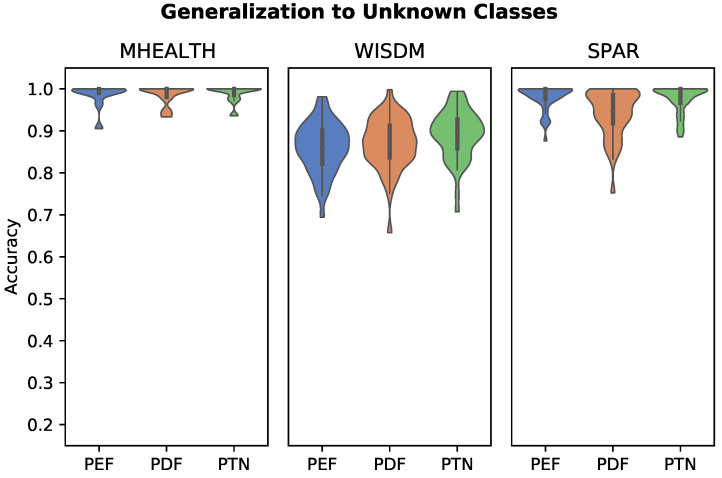
Distribution of activity classification performance when generalizing an embedding to novel activity classes, with 30% of activity classes held back from the training set. The PTN model achieved the highest mean accuracy across all three datasets.

Results of generalization to new activity class experiments are plotted in Figure 6. Results are similar to in-distribution classification tasks, with all three feature classifiers achieving near perfect performance, with the exception of the WISDM dataset. The PTN algorithm achieved the highest accuracy across all three datasets, though these results are again with standard deviation of one another.

### 3.6. Computational Expense

The computational cost for each model on the SPAR dataset is reported in Table 3, detailing training and inference time on our hardware, and storage size for model and reference data. In our implementation, the inference time for the PDF and PTN classifiers was split nearly equally between embedding computation and nearest embedding search. Training the FCN core with triplet loss in the PTN model increased the fit time by approximately five-fold in comparison to training with categorical cross entropy loss as with the PDF and FCN models.

## 4. Discussion

This work describes the methodology and use of novel approaches to personalized human activity recognition of inertial data. A personalized deep feature model (PDF), a personalized triplet network (PTN), and personalized engineered features (PEF) were compared to a baseline impersonal fully convolutional network (FCN).

The PTN and PDF models outperformed PEF for activity classification. The three personalized feature classifiers significantly outperformed the impersonal FCN classifier, which is considered a strong baseline. In fact, the personalized classifiers were able to achieve performance approaching training set performance of the impersonal FCN classifier, nearing 100% mean accuracy in cross-validated classification. However, as the reference and test sets for the personalized classifier evaluation were obtained by splitting individual time series (without temporal overlap), our results likely overestimate real-world performance where the reference and test sets would be derived from separate physical therapy sessions.

Within the spectrum of personalized algorithms evaluated here there are some notable differences in performance. The PTN with single subject triplet loss as proposed in this work not only achieves the highest classification accuracy, standard deviation is also much more constrained around the mean, and is a marked improvement even over the PTN ${}^{†}$ algorithm, which implemented a naive splitting strategy. The FCN classifier performed poorly for some individuals (as low as 50% accuracy), as shown in Figure 2. The three personalized feature classifiers evaluated all significantly mitigated inter-subject variability in terms of accuracy of prediction, and exhibited more consistently accurate predictions for individual subjects within each dataset.

Experimental results of algorithms on the WISDM sets were comparatively poor for classification tasks versus the MHEALTH and SPAR datasets. While the WISDM dataset was unique in being segmented with a 10-second time window, we believe that unlikely to be the source of this discrepancy, as we have previously evaluated window size and found only a moderate effect on accuracy across several datasets [34]. Instead, this effect is likely the result of the selection of activities of daily living in the WISDM dataset, which are confused due to extremely similar patterns in the inertial data from a single wrist IMU (e.g., eating soup, eating chips, eating pasta, and eating sandwich).

The novel triplet loss function (Equation (2)) and triplet selection strategy described in this work significantly improved the performance of the PTN model in comparison to conventional triplet loss. The subject triplets can be considered “hard” triplets in the context of other strategies for specifically selecting hard triplets to improve TNN training [11,51,52,53]. How well our approach compares to other hard triplet selection strategies remains as future work. However, our strategy may be worth considering as it is straightforward to implement and computationally inexpensive in comparison to strategies that require embeddings to be computed prior to triplet selection. The benefit of subject triplets may hold to a greater extent on datasets collected with heterogenous hardware. Certainly, our work demonstrates that the triplet selection method is an important consideration for maximizing the utility of TNNs in the inertial activity recognition context.

Dependence of model performance on reference dataset size and embedding size were explored. Performance of PTN and PDF models appear robust to smaller embedding size, whereas the PEF model experienced a significant drop in accuracy at embedding sizes 16 and below. Twenty-four reference segments were selected as the upper limit for the effect of the reference data size experiment. This upper bound is constrained by recording length in the SPAR dataset, but based on these results, additional reference segments may improve accuracy for longer recordings. The results showed that performance suffered significantly when using a reference size of four segments. This could partially be an effect of having fewer reference segments than the number of activity classes, thereby creating a k-NN training set that may not include any segments from the same class as the test segment. Based on the results in Figure 4, 16 reference segments (equal to approximately 16 seconds of data) or more should be used per activity class.

Typically, deep learning classification algorithms implementing a softmax output layer perform poorly at out-of-distribution activity detection due to overconfidence [54]. Various approaches to improving OOD performance for neural networks have been investigated in the computer vision fields with mixed results and this remains an active area of research [33]. An advantage of using personalized features for activity classification is the built-in capability to use them for OOD activity detection and classification of novel activities. In the HAR field, OOD detection is particularly important as there exists an infinite number of possible human actions, and therefore it may be impractical to include all possible actions in the training set or even all reasonably likely actions. Typically, it is a desirable property of an HAR system that it can be trained to recognize a select number of pertinent activities and have the ability to reject anomalous activities not in the training distribution.

In these experiments, personalized models significantly outperformed the baseline FCN softmax threshold OOD detection method in the WISDM dataset, unlike for the MHEALTH or SPAR datasets where performance was roughly equivalent. Superior performance in comparison to a softmax threshold OOD method would be expected, given existing work on OOD detection in exercise IMU datasets [34]. While the activities included in MHEALTH and SPAR are exercise and full-body movements, WISDM includes a larger number (18) of activity classes, including a number of very similar activities of daily living (as previously noted). OOD detection of WISDM in these experiments is thus a more challenging problem, particularly when an OOD activity in the test set is nearly identical in terms of inertial data patterns to the patterns of one or more in-distribution activities used to train the model. Our results show that the personalized models, in particular the PTN, significantly outperformed softmax thresholding in these cases. This suggests that the PTN may be suited to HAR OOD-detection problems where there is greater inter-patient heterogeneity than inter-activity heterogeneity. Unlike in classification experiments where personalized algorithms achieved near perfect accuracy, OOD detection accuracy was significantly lower. In contrast to classification tasks, for OOD, the k-NN of personalized methods is never trained on reference samples from the selected OOD classes, and depends rather on a threshold-based distance metric for prediction of untrained classes.

We have demonstrated that mean nearest neighbor distance with personalized features has good performance for our synthetic OOD evaluation. However, further work is required to evaluate alternative approaches and build out-of-distribution datasets incorporating real-world variation with unknown and potentially unsupervised daily activities.

Personalized models have the flexibility to be generalized to new activity classes, provided that a reference recording from the new class is available from the patient in question. The PEF, PDF, and PTN models achieved generalization performance similar to their performance when trained on the full set of exercises in the previous classification tasks. This demonstrates the ability of these personalized models to effectively generalize to new activity classes with very little new data.

While the PTN model exhibited competitive performance, a significant disadvantage of using a triplet neural network to learn the embedding function is the increased computational cost during training. On our hardware, the PTN approach increases the training time five-fold and triples the GPU memory requirements in comparison to training an identical core with categorical cross entropy loss. This is due to the further cost of triplet selection where each triplet is comprised of three distinct samples that must each be embedded to compute the triplet loss. Fortunately, once the embedding has been trained, there is little difference in computational requirements to compute the embedding or classify an unknown sample.

The FCN core architecture described in this work, with just 278,848 parameters (∼1 MB), is a relatively compact model. Particularly, in comparison to computer vision or language models that can exceed tens or hundreds of millions of parameters [55,56,57]. Given the small size of the model and reference embeddings, implementing a personalized feature classifier based on the FCN core may be feasible within an edge computing system where the computations for HAR are performed locally on the user’s hardware (mobile device). There are various advantages of an edge computing approach, including improved classification latency, reliability, and network bandwidth usage [58].

The personalized k-NN model used to search reference embeddings for classification of test samples in the PEF, PDF, and PTN models was found to be effective, but approximately doubles the inference time in comparison to the FCN model that used a softmax layer for classification. A disadvantage with k-NN search is that computational time complexity and data storage requirement scales with the number of reference samples O(N). This property of k-NN limits its utility as an impersonal classifier, as performing inference requires searching the entire training dataset. In the context of a personalized algorithm, however, the k-NN search is limited only to the subject’s reference samples, which we have demonstrated need only include tens of samples per activity class. Of course, other search strategies could be implemented to search the reference data. The nearest centroid method, for instance, could be used which has computational complexity O(1), scaling linearly with the number of reference classes.

Although there was no temporal overlap in the segments used to derive the reference and test embeddings, it is a limitation of this work that they were derived from the same time series. Unfortunately, we are not aware of any currently available public inertial activity recognition datasets that contain repeated data collections of the same activity classes by the subjects. Certainly, such a dataset would be worthwhile to collect and would serve as the best validation of the approaches described in this work. However, these experimental results illustrate that personalized algorithms are an effective approach to reducing inter-subject algorithm performance variability, which is one of the key motivations for this research. As such, we would expect personalized algorithms to exhibit better performance than impersonal classifiers such as the FCN when tested on a dataset with repeated data collections of the same activity classes. Similarly, since the PTN appears the most effective model for reducing inter-subject variability, we believe this provides strong evidence for the superior performance of the PTN model versus the other personalized algorithms implemented in these experiments.

## 5. Conclusions

We have shown that the personalized algorithms presented here are more robust to inter-subject variability in inertial time series datasets. They significantly outperform impersonal approaches in more challenging classification tasks where there exists a high degree of similarity between classes (e.g., WISDM). This is especially apparent for OOD detection where the OOD data is similar to in-distribution class training data. These algorithms also have built-in functionality for generalization to new activity classes. We have, furthermore, presented a novel single subject triplet loss, which improves subject-specific prediction performance over both a naive triplet loss implementation as well as the other personalized algorithms evaluated. This method is also shown to significantly reduce inter-subject variability in activity classification tasks. These algorithms should be further evaluated on a dataset containing multi-session performance of exercises by each subject for validation in a realistic use case scenario. Nevertheless, we believe these results present strong evidence that the personalized algorithms as presented here, and, in particular, the PTN improves detection and classification accuracy through focused learning of the heterogeneous data of individual subjects.

## Figures and Tables

**Table 1:** Experimental inertial datasets.

Dataset	Sensors	Subjects	Classes ${}^{1}$	Sampling	Omitted Subjects	Domain	Sensor Placement
MHEALTH	9-axis IMU${}^{2}$ x3, 2-lead ECG	10	12	100 Hz	0	Exercise	Chest, left ankle, right arm
WISDM	6-axis IMU x2	51	18	20 Hz	4	ADL, Exercise	Right pant pocket, wrist
SPAR	6-axis IMU x1	40	7	50 Hz	0	Physiotherapy	Wrist

^1^ The following activities were performed in each dataset. MHEALTH: Standing still, sitting, lying down, walking, climbing stairs, waist bends forward, frontal elevation of arms, knees bending, cycling, jogging, running, jump front and back. WISDM:Walking, jogging, ascending/descending stairs, sitting, standing, kicking a soccer ball, dribbling a basketball, catching a tennis ball, typing, writing, clapping, brushing teeth, folding clothes, eating pasta, eating soup, eating a sandwich, eating chips, drinking from a cup. SPAR: Pendulum, abduction, forward elevation, internal rotation with resistance band, external rotation with resistance band, lower trapezius row with resistance band, bent over row with 3 lb dumbell.

**Table 2:** Activity classification performance ${}^{1}$.

Model	MHEALTH	WISDM	SPAR
FCN	0.925 ± 0.049	0.754 ± 0.012	0.947 ± 0.069
PEF	0.984 ± 0.029	0.852 ± 0.060	0.971 ± 0.038
PDF	0.995 ± 0.016	0.889 ± 0.055	0.980 ± 0.028
PTN ${}^{†}$	0.993 ± 0.024	0.909 ± 0.054	0.978 ± 0.035
PTN	**0.999** ± **0.003**	**0.913** ± **0.053**	**0.990 ± 0.017**

^1^ Classification performance of the fully-convolutional neural network (FCN), personalized engineered feature model (PEF), personalized deep feature model (PDF), personalized triplet network trained with conventional triplet loss (PTN^†^), and the personalized triplet model trained with patient-specific triplet loss (PTN). Scores are the cross-validated classification accuracy (mean ± standard deviation) aggregated by subject. ^†^ The PTN trained with conventional triplet loss.

**Table 3:** Computational and storage expense.

Model	Fit Time [s]	Inference Time [s]	Model Size [kB]	Reference Size [kB]
FCN	137	0.47	4290	0
PEF	3.3	0.39	3.8	112
PDF	129	0.94	1095	112
PTN	667	1.3	1095	112

## Data Availability

The MHEALTH (accessed on 15 November 2019), WISDM (accessed on 15 November 2019), and SPAR (accessed on 15 November 2019) datasets used in this study are open-source and freely available. The Keras code used to create the FCN model is available here (accessed on 15 November 2019).

## Abbreviations

The following abbreviations are used in this manuscript:

HAR	Human activity recognition
TNN	Triplet neural network
OOD	Out-of-distribution
SNN	Siamese neural network
FCN	Fully-convolutional neural network
ReLU	Rectified linear unit
PEF	Personalized engineered feature
PDF	Personalized deep feature network
PTN	Personalized triplet network
k-NN	k-Nearest Neighbors
IMU	Inertial measurement unit
ADL	Activities of daily living
AUROC	Area under the receiver operator characteristic curve
